# Open Access Publishing

**DOI:** 10.3332/ecancer.2011.ed11

**Published:** 2011-09-01

**Authors:** Linda Cairns, William Russell-Edu

**Affiliations:** 1 Science Editor, ecancermedicalscience, European Institute of Oncology, Milan, Italy; 2 Librarian, European Institute of Oncology, Milan, Italy

A leading comment in yesterday’s Guardian, George Monbiot’s provocatively titled and aggressively argued Academic publishers make Murdoch look like a socialist is bound to irritate some of the big academic publishing companies, but the time has come to bring this argument to the attention of all.

Open Access (OA) is very much a hot topic among researchers and librarians, and this stirring article raises many interesting points. OA refers to unrestricted online access to articles published in scholarly journals and has been fueled by instant connection to the World Wide Web, thus allowing low-cost distribution of academic material. However, academic publishers have a lot to lose financially.

As Monbiot states in his piece, “people should be encouraged to understand science and other academic research. Without current knowledge, we cannot make coherent democratic decisions. But the publishers have slapped a padlock and a ‘keep out’ sign on the gates.”

Prices of academic journals are often prohibitively expensive. Monbiot specifically cites Elsevier, a major publisher in the field, as their *Biochimica et Biophysica Actacosts* $20,930 per year, but they are by no means the only publishers who put stress on a librarian’s budget.

The article reports that downloading one, single paper can cost up to $42. Recently I have encountered papers costing up to $100. Elsevier, Springer, Wiley and others are laughing all the way to the proverbial bank. As is pointed out, the notorious Robert Maxwell made much of his millions from academic publishing.

At least the infamous Rupert Murdoch pays those who write for his publications. Academic publishers receive papers from their writers for free, while the taxpayers funded the research and then have to pay exorbitant costs again to see the results of the research.

It is little wonder that many people rely on informal networks of friends and colleagues to collect papers necessary for their research. I have even heard of librarians breaching copyright law in this way.

I recall once, in a moment of extreme deadline desperation, asking a librarian in the US if he could, off the record, send me a paper published by someone in their university; however, his reply only just stopped short of threatening me with legal action.

Open Access has so far “failed to displace the monopolists”, as Elsevier are still riding high on a 40% profit margin. Monbiot calls for academic publishers’ practices to be brought to the attention of the competition watchdogs and encourages publicly-funded institutional repositories; whereby research articles can be stored and “liberated”.

A recent paper from the European Association of Cancer Research (EACR), which will appear next week in *e*cancermedicalscience, an open access journal in which it is free to publish, discusses in detail the effect of publishing costs and access to information on cancer research. The results of an EU funded project highlighted the issue of access to subscription journals, the barrier to essential and urgent information that a ‘paywall’ creates, and the need for free access.

The authors were amazed to find that a quarter of cancer researchers experience difficulty gaining access to what their peers have published, and that they are compelled to spend valuable time tracking down and supplying themselves with such publications without them costing an arm and a leg. It is especially salient for those working independently without institutional library back-up. Publishers frown on them using “informal networks of friends and colleagues” but if a paper can only be downloaded at the all too common cost of $100, what are the realistic options?

“Journal prestige” is a big issue, not just for personal ego, but publishing in a high-impact factor, prestigious, journal is pretty much demanded by most institutions for tenure and funding. As the authors say, “if OA can address any remaining concerns over esteem and quality authors will progressively move across to this model”. It will take time for an OA journal to gain the prestige of Nature, but what if Nature were to become open access?

“Eighty-eight percent of authors believe that publicly funded research should be made available to be read and used without access barriers” and the statistics speak for themselves. Perhaps publishers fear they’ll suffer like the music industry with, less than legal, rampant digital downloads, but “change is inevitable”.

Bearing in mind that this report was requested by the EC, some action is necessary.

The huge number of comments on the Guardian website provides an interesting insight “from the coal face” and offer many starting points for a stimulating discussion and debate also here on *e*cancermedicalscience’s site.

